# Long journey of 16S rRNA‐amplicon sequencing toward cell‐based functional bacterial microbiota characterization

**DOI:** 10.1002/imo2.9

**Published:** 2024-07-11

**Authors:** Jianshi Jin, Xiongduo Liu, Katsuyuki Shiroguchi

**Affiliations:** ^1^ State Key Laboratory of Integrated Management of Pest Insects and Rodents, Institute of Zoology Chinese Academy of Sciences Beijing China; ^2^ Laboratory for Prediction of Cell Systems Dynamics RIKEN Center for Biosystems Dynamics Research (BDR) Osaka Japan

**Keywords:** 16S rRNA‐amplicon sequencing, absolute quantification, bacterial microbiota, high‐throughput measurement, single cell analysis

## Abstract

Bacteria often exist and function as a community, known as the bacterial microbiota, which consists of vast numbers of bacteria belonging to many bacterial species (taxa). Characterizing the bacterial microbiota needs high‐throughput approaches that enable the identification and quantification of many bacterial cells, and such approaches have been under development for more than 30 years. In this review, we describe the history of high‐throughput technologies based on 16S ribosomal RNA (rRNA) gene‐amplicon sequencing for the characterization of bacterial microbiotas. Then, we summarize the features and applications of current 16S rRNA gene‐amplicon sequencing approaches, including a recent achievement that enables the identification of individual cells with single‐base accuracy for 16S rRNA genes and the quantification of many identified cells. Furthermore, we present the prospects for further technical development, including the combined use of high‐throughput methods and other informative analyses, such as whole‐genome sequencing in the common unit of the cell, which enables bacterial microbiota characterization based on both the number of cells and their functions.

## Highlights

- We summarized the history of 16S ribosomal RNA (rRNA) gene‐amplicon sequencing approaches.
- We clarified the features of current 16S rRNA gene‐amplicon sequencing approaches.
- Recent development enables the quantification of cells for many identified bacterial types.
- We discussed the new era of cell‐based functional bacterial microbiota characterization.

## Graphical abstract

In this review, we first summarized the history of 16S ribosomal RNA (rRNA) gene‐amplicon sequencing approaches. Then, we clarified the features of current 16S rRNA gene‐amplicon sequencing approaches. Importantly, recent development enables the quantification of cells for many identified bacterial types. Finally, we discussed the new era of cell‐based functional bacterial microbiota characterization.

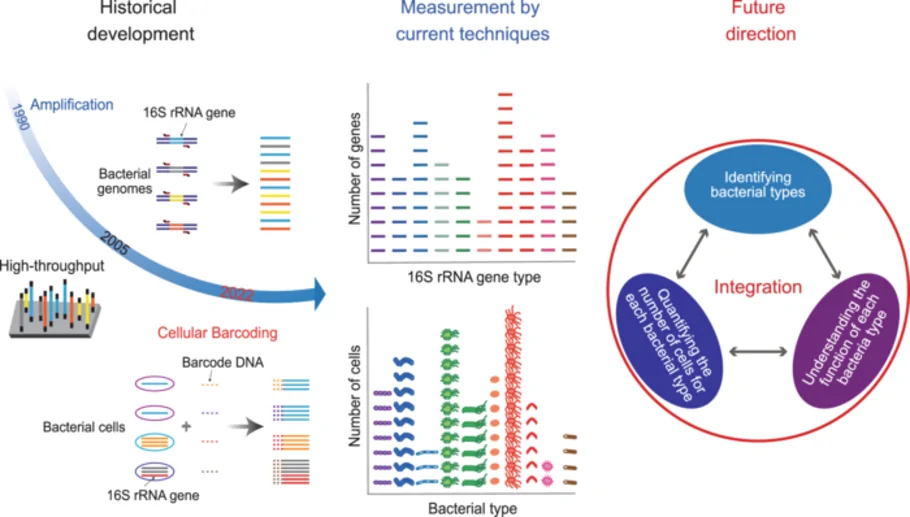

## 1 INTRODUCTION

Bacteria live as communities known as bacterial microbiotas in marine and terrestrial environments either independently or symbiotically with other organisms, such as humans, other animals, and plants [1, 2, 3, 4]. Recently, there has been increased interest in the manipulation of environmental bacterial microbiotas to restore ecosystem functions [5] and the manipulation of the commensal bacterial microbiota to restore host health [6, 7, 8]. To understand how the bacterial microbiota interacts with the environment and other organisms and to manipulate the microbiota to control their interactions, the primary question of “What constitutes the bacterial microbiota?” should be addressed. Indeed, compositional analysis still involves the fundamental question: What types of bacteria (i.e., taxa) and how many organisms (i.e., bacterial cells) of each taxon are included in the bacterial microbiota?

The comparative analysis of genomic sequences is a powerful approach for identifying and classifying bacterial types since a bacterial cell's genome is the ultimate record of its evolutionary history [9]. However, it is not practically possible to determine the whole‐genome sequences of all bacterial cells in the microbiota (i.e., the microbiome), even using state‐of‐the‐art technologies. Therefore, there is a trade‐off between how many bases in the genome per cell and how many cells can be measured in a single experiment. Although determining many different genes for a bacterial microbiota by whole‐genome sequencing or metagenomics methods could obtain gene‐based functional compositional insights [10, 11, 12], to perform high‐throughput analysis of many bacterial cells, the analysis of one gene or even a part of a gene per cell is a suitable approach [13]. For this purpose, the 16S ribosomal RNA (rRNA) gene (approximately 1500 base pairs) has been widely used as a gene marker for classifying bacteria in phylogenetic studies since 1977 [9] because the 16S rRNA gene has the following features: (1) it is ubiquitously present in all bacterial cells; (2) it contains nine hypervariable regions (V1−V9, each with approximately 30−100 base pairs) that are assumed to vary during evolution and can be used to efficiently distinguish bacterial species [9, 14]; and (3) there are highly conserved regions between the nine hypervariable regions among bacteria, which can be used as targets for the purification or amplification of 16S rRNA gene‐coded DNA molecules [14]. To experimentally characterize bacterial microbiota using 16S rRNA genes, sequencing methods for the identification of 16S rRNA genes from microbiotas have been in development since 1985 [15, 16]. In most developed 16S rRNA gene‐amplicon sequencing methods, the 16S rRNA genes in bacteria are amplified using a pair of primers targeting the highly conserved regions, and then the amplified DNA molecules are sequenced using a high‐throughput sequencing platform.

The high‐throughput 16S rRNA gene‐amplicon sequencing method also has the following advantages: since its measurement relies on the amplification of the 16S rRNA genes, it is unlimited for unculture bacteria and low‐bacteria‐biomass sample and is bacteria‐specific in mixed samples. Therefore, this method is currently being used in a variety of fields, such as medicine, agriculture, and environmental research. In medicine, the 16S rRNA gene‐amplicon sequencing is used to identify specific pathogens in oropharyngeal and rectal samples for analyzing epidemic and infectious diseases [17, 18], to detect specific bacteria from low‐bacteria‐biomass samples, such as blood and tumors, for studying blood diseases [19] and cancers [20, 21], and to characterize microbiotas in cerebrospinal fluid and respiratory samples as a reference for developing an antimicrobial treatment plan [22, 23]. In agriculture, the 16S rRNA gene‐amplicon sequencing is used to detect pathogens in fresh produce for controlling food safety [24, 25] and to explore microbiota on plants for studying microbiota−plant interaction, which is highly related to plant growth [26], plant resistance [27], and plant uptake of soil nutrients [28]. In environmental research, 16S rRNA gene‐amplicon sequencing is used to profile the microbiota composition of environment samples, such as wastewater and lake water, for monitoring environmental pollution [29, 30] and the effect of ecological restoration [31].

In this review, we summarize the features of widely used and recently developed 16S rRNA gene‐amplicon sequencing methods employed for the identification and quantification of 16S rRNA genes or bacterial cells, starting from the historical milestones where important measurement concepts were verified. Then, we summarize the recent progress and expanded applications of 16S rRNA sequencing approaches. Finally, we discuss the future of 16S rRNA gene‐amplicon sequencing for achieving an in‐depth understanding of the bacterial microbiota.

## 2 MILESTONES OF 16S rRNA GENE‐AMPLICON SEQUENCING

**Figure 1:**
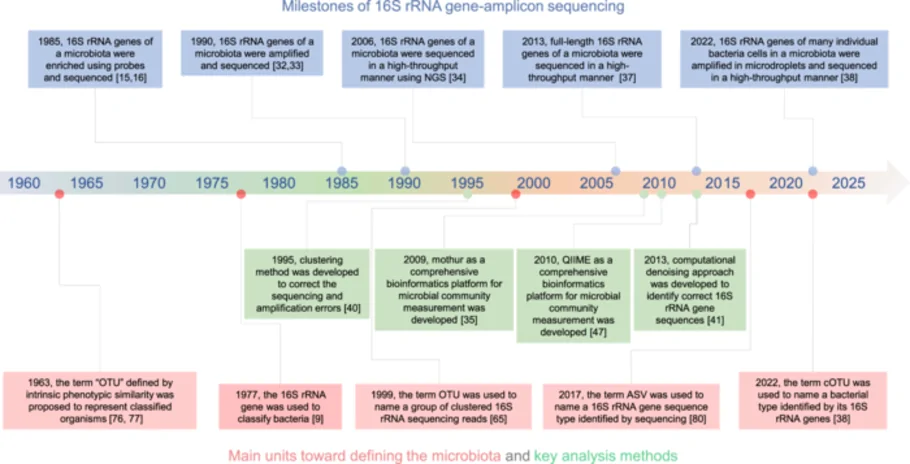
History of 16S rRNA gene‐amplicon sequencing, key analysis methods, and related classification units. ASV, amplicon sequence variants; cOTU, cell‐based operational taxonomic unit; NGS, next‐generation sequencing; OTU, operational taxonomic unit; QIIME, quantitative insights into microbial ecology; rRNA, ribosomal RNA.

Here, we summarize the conceptual achievements in 16S rRNA gene‐amplicon sequencing technology (Figure 1) that established the currently used 16S rRNA gene‐amplicon sequencing approaches. In 1985, the characterization of the composition of a bacterial community (or microbiota) via the identification of 16S rRNA gene sequences without cell culture was first successfully achieved by Pace et al. [15, 16], which gave rise to the era of whole‐microbiota characterization. Under this approach, the 16S rRNA genes of a bacterial community are enriched by targeting universally conserved regions between the hypervariable regions of 16S rRNA genes using DNA probes, which are then amplified by DNA cloning and sequenced by Sanger sequencing. In 1990, a more selective method applied before cloning was combined with this approach, in which 16S rRNA genes are specifically amplified by polymerase chain reaction (PCR) using a pair of primers [32, 33] targeting the conserved regions [14]. This selective amplification step is essential for efficiently enriching the 16S rRNA genes of microbiota that exist alongside other organisms in complex habitats, and this selective amplification is still a key step in the current 16S rRNA gene‐amplicon sequencing approach. In 2006, a next‐generation sequencing technique that enables massively parallel sequencing was first used for 16S rRNA gene‐amplicon sequencing [34]. Although next‐generation sequencing‐based approaches can determine the sequences of only a portion of 16S rRNA genes (at present, up to 600 bases can be sequenced), they have dramatically increased the throughput of measurable 16S rRNA genes by several orders of magnitude [35]. This is very important for bacterial microbiota characterization since microbiotas contain a large number of bacteria. For example, there are approximately 10^11^ bacterial cells per gram in human feces [36]. In addition, the DNA cloning step is not required for this approach [34], which simplifies the protocol. At present, next‐generation sequencing‐based approaches are largely dominating 16S rRNA gene amplicon sequencing. In 2013, the 16S rRNA gene‐amplicon sequencing approach was expanded to a single‐molecule long‐read sequencing platform [37], which allowed the analysis of full‐length 16S rRNA genes in a high‐throughput manner. In 2022, barcoding bacteria for identification and quantification (BarBIQ), a single cell‐based 16S rRNA gene‐amplicon sequencing approach using cellular barcoding, was developed, through which the types of bacterial cells in microbiota can be identified based on their 16S rRNA genes in a high‐throughput manner [38, 39]. BarBIQ also successfully quantified the cell number of each identified bacterial type in the microbiota.

Along with the experimental developments above, analysis methods have also been developed to improve the performance of the 16S rRNA gene‐amplicon sequencing approach that amplifies 16S rRNA genes from a microbiota by correcting sequencing and amplification errors. As far as we know, the first analysis method was developed in 1995 [40] that corrected the sequencing and amplification errors using a sequence‐based clustering approach. Later, many different sequencing‐based clustering approaches have been developed (see below) and are widely used even now (Figure 1). However, the clustering process is influenced by coexisting sequences in the same dataset, making it difficult to compare the results from different studies or samples. Therefore, computational denoising approaches were developed in 2013 to identify correct 16S rRNA gene sequences from sequencing reads independently without clustering (Figure 1) [41, 42, 43, 44, 45, 46]. Furthermore, to integrate different analysis methods to comprehensively analyze the data generated from 16S rRNA gene‐amplicon sequencing, platforms such as mothur [35] and quantitative insights into microbial ecology (QIIME) [47] were established in 2009 and 2010 (Figure 1) and have been widely used since then, which has promoted the use of a worldwide standard format to present results.

## 3 IDENTIFICATION OF 16S rRNA GENES

Identification of the taxa of the bacteria (bacterial types) present in microbiota is a main goal of 16S rRNA gene‐amplicon sequencing approaches. However, most approaches identify only 16S rRNA gene sequence types, which do not directly represent bacterial types since a bacterial cell may contain multiple 16S rRNA gene sequence types [14, 48]. In this section, we describe several currently used approaches for identifying 16S rRNA gene sequence types with recent improvements.

### Current approaches for the identification of 16S rRNA genes

The current widely used 16S rRNA gene‐amplicon sequencing approaches are all based on next‐generation and single‐molecule sequencing strategies that enable high‐throughput analysis at low cost. However, with these methods, it is difficult to accurately identify 16S rRNA gene sequence types due to considerable sequencing and amplification errors (e.g., chimeras [49]) [14, 50]. To correct sequencing and amplification errors, additional approaches such as sequence‐based clustering, computational denoising, and molecular barcoding have been developed in recent decades.

First, in the most commonly used 16S rRNA gene‐amplicon sequencing approach in recent decades, 16S rRNA genes are amplified from a bacterial microbiota (i.e., a pool of many bacteria) and sequenced, and the sequenced 16S rRNA gene sequences (i.e., sequencing reads) are then clustered into groups based on sequence identity using a certain threshold and different clustering algorithms (Table 1) [48]. Finally, all sequencing reads in the same clustered group are annotated according to a single representative 16S rRNA gene sequence [48]. The concept of this clustering‐based approach is based on the idea that taxonomically similar organisms have similar 16S rRNA gene sequences [14, 48, 63] and that sequencing or amplification errors in the sequencing reads are minor. Under this clustering‐based approach, 16S rRNA gene types that have similar sequences cannot be distinguished.

**Table 1:** Comparison among different 16S rRNA gene‐amplicon sequencing approaches.

Experimental approach	Data processing approach			Identification	Length of identified 16S rRNA gene sequence	Database dependence for identification	Quantification	Relative/absolute quantification	Comparison of results in the same approach^a^
	Approach name	Software and algorism	Taxonomic unit						
**16S rRNA gene‐amplicon sequencing approach that amplifies 16S rRNA genes from a microbiota (i.e., a pool of many bacteria)**	de novo OTU approach [51, 52]	**mothur** (https://mothur.org/): 16S rRNA sequencing reads are clustered by either the furthest, average, or nearest neighbor algorithm using the software DOTUR or clustered by VSEARCH algorithm with a user‐defined threshold [35]. **USEARCH** (https://drive5.com/uparse/): 16S rRNA sequencing reads are clustered by UPARSE‐OTU algorithm using the software USEARCH with a user‐defined threshold [53]. **Quantitative insights into microbial ecology (QIIME2)** (https://qiime2.org/): 16S rRNA sequencing reads are clustered by greedy algorithm using the software VSEARCH with a user‐defined threshold [54].	Operational taxonomic unit (OTU)	16S rRNA gene sequences	Partial	Independent	The number of amplified molecules for each identified 16S rRNA gene sequence	Relative	Incomparable (need reprocessing of combined data for comparison)
	Closed‐reference OTU approach [51, 52]	**mothur** (https://mothur.org/) [35]. **USEARCH** (https://drive5.com/uparse/) [53]. **QIIME2** (https://qiime2.org/) [54].	OTU	16S rRNA gene sequences	Partial	Dependent	The number of amplified molecules for each identified 16S rRNA gene sequence	Relative	Incomparable (need reprocessing of combined data for comparison)
	Open‐reference OTU approach [51, 52]	**mothur** (https://mothur.org/) [35]. **USEARCH** (https://drive5.com/uparse/) [53]. **QIIME2** (https://qiime2.org/) [54].	OTU	16S rRNA gene sequences	Partial	Dependent	The number of amplified molecules for each identified 16S rRNA gene sequence	Relative	Incomparable (need reprocessing of combined data for comparison)
	Computational denoising approaches	**QIIME2** (https://qiime2.org/): the correct and incorrect sequencing reads are distinguished by statistical analysis based on the error rates of sequencing and amplification using the software DADA2, or based on an upper error rate bound along with a constant probability of indels and the mean read error rate using the software Deblur [54].	Amplicon sequence variant (ASV)	16S rRNA gene sequences	Partial	Independent	The number of amplified molecules for each identified 16S rRNA gene sequence	Relative	Directly comparable
		**USEARCH** (https://www.drive5.com/usearch/manual/unoise_algo.html): the correct and incorrect sequencing reads are distinguished by determining an abundance threshold using the software Unoise3 [55].	Zero‐radius operational taxonomic unit (zOTU)	16S rRNA gene sequences	Partial	Independent	The number of amplified molecules for each identified 16S rRNA gene sequence	Relative	Directly comparable
**16S rRNA gene‐amplicon sequencing approach that amplifies 16S rRNA genes with molecular barcoding** [56, 57, 58]	Closed‐reference OTU approach [51, 52]	**mothur** (https://mothur.org/) [35]. **USEARCH** (https://drive5.com/uparse/) [53].	OTU	16S rRNA gene sequences	Partial	Dependent	The number of intact 16S rRNA gene molecules for each identified 16S rRNA gene sequence	Relative and absolute	Incomparable (need reprocessing of combined data for comparison)
**16S rRNA gene‐amplicon sequencing approach that amplifies 16S rRNA genes from each bacteria cell using droplets and cellular barcodes** [38, 39]	BarBIQ [38, 39]	**BarBIQ_Pipeline** (https://github.com/Shiroguchi-Lab/BarBIQ_Pipeline_V1_2_0): sequencing reads are first clustered using cellular barcodes, and then the 16S rRNA sequencing linked to the same cellular barcode are further clustered based on their sequence identities.	Cell‐based operational taxonomic unit (cOTU)	Bacterial types (cOTUs) based on 16S rRNA sequences with single‐base accuracy	Partial	Independent	The number of bacterial cells for each identified bacterial type (cOTU)	Relative and absolute	Directly comparable
**16S rRNA gene‐amplicon sequencing approach that amplifies full‐length 16S rRNA genes from a microbiota (i.e., a pool of many bacteria)**	Closed‐reference OTU approach [51, 52]	**MetaMaps** (https://github.com/marbl/MashMap): full‐length 16S rRNA sequencing reads are clustered by expectation–maximization algorithm using the software MetaMaps [59]. **Emu** (https://gitlab.com/treangenlab/emu): full‐length 16S rRNA sequencing reads are clustered by expectation–maximization algorithm using the software Emu [60].	OTU	16S rRNA gene sequences	Full‐length	Dependent	The number of amplified molecules for each identified 16S rRNA gene sequence	Relative	Incomparable (need reprocessing of combined data for comparison)
	Open‐reference OTU approach [51, 52]	**USEARCH** (https://drive5.com/uparse/) [53]. **NanoCLUST** (https://github.com/genomicsITER/NanoCLUST): full‐length 16S rRNA sequencing are clustered by HDBSCAN (Hierarchical Density‐Based Spatial Clustering of Applications with Noise) algorithm by using the software NanoCLUST [61]. **16S full‐length amplicon sequencing** **data analysis software (16S‐FASAS)** (https://github.com/capitalbio-bioinfo/FASAS): full‐length 16S rRNA sequencing are clustered by MegaBLAST tools by software 16S‐FASAS [62].	OTU	16S rRNA gene sequences	Full‐length	Dependent	The number of amplified molecules for each identified 16S rRNA gene sequence	Relative	Incomparable (need reprocessing of combined data for comparison)
	Computational denoising approaches	**QIIME2** (https://qiime2.org/) [54]. **USEARCH** (https://www.drive5.com/usearch/manual/unoise_algo.html) [55].	ASV zOTU	16S rRNA gene sequences	Full‐length	Independent	The number of amplified molecules for each identified 16S rRNA gene sequence	Relative	Directly comparable

Abbreviations: DOTUR, distance‐based OTU and richness; rRNA, ribosomal RNA.

^a^ This column indicates that the results separately processed from the sequencing data using the same approach can be directly compared or not.

To our knowledge, this clustering‐based approach was used for the first time in 1995 [40] and later in 1999 [64, 65, 66] and has been continuously used since then, which extends across almost the whole history of 16S rRNA gene‐amplicon sequencing methods based on Sanger sequencing, next‐generation sequencing, and single‐molecule long‐read sequencing (Figure 1). In 2001, a review paper chose the term operational taxonomic unit (OTU) to describe the group of clustered 16S rRNA sequencing reads for the explanation of all reviewed studies [67]. Later, the term OTU was used in several analytical software programs, such as distance‐based OTU and richness (DOTUR) [68], USEARCH [53], mothur [35], QIIME [47, 54], and EasyAmplicon [69], and this term has now become the worldwide standard terminology for the group of clustered 16S rRNA sequence reads in the 16S rRNA gene‐amplicon sequencing approach.

Basically, there are three OTU‐based approaches, and they are well documented in reviews [51, 52]: the de novo OTU approach, the closed‐reference OTU approach, and the open‐reference OTU approach (Table 1). In the closed‐reference and open‐reference OTU approaches, databases are essential for OTU identification, particularly for less‐studied samples. Recently, databases, such as Greengenes2 [70], SILVA (updated to 2020) [71], microbial database for activated sludge 4 (MiDAS 4) [29], MetaSquare [72], RiboGrove [73], forensic microbiome database [74], and comprehensive ecosystem‐specific reference databases [75], were developed to improve OTU identification for each specific sample or to integrate 16S rRNA gene databases with bacterial whole‐genome databases.

The term OTU was first used in 1963, which represents a group of organisms defined by intrinsic phenotypic similarity [76, 77] (we refer to this OTU as “OTU”). This “OTU” is different from the OTU above in the 16S rRNA gene‐amplicon sequencing approaches, which represent a group of 16S rRNA sequencing reads generated by clustering based on sequence identity [48, 51, 68, 78, 79]. Because some bacteria have multiple 16S rRNA sequence types in their genomes and the multiple 16S rRNA sequence types are sometimes significantly different (sequence identity <95%) [41, 48], the 16S rRNA sequences of a given bacterial organism may be clustered into different OTUs. Therefore, the OTUs identified in the 16S rRNA gene‐amplicon sequencing methods, which represent sequence types, are essentially different from the original “OTUs,” which define organisms. Thus, the OTU‐based approach is not an efficient tool to classify bacterial organisms based on 16S rRNA genes.

**Figure 2:**
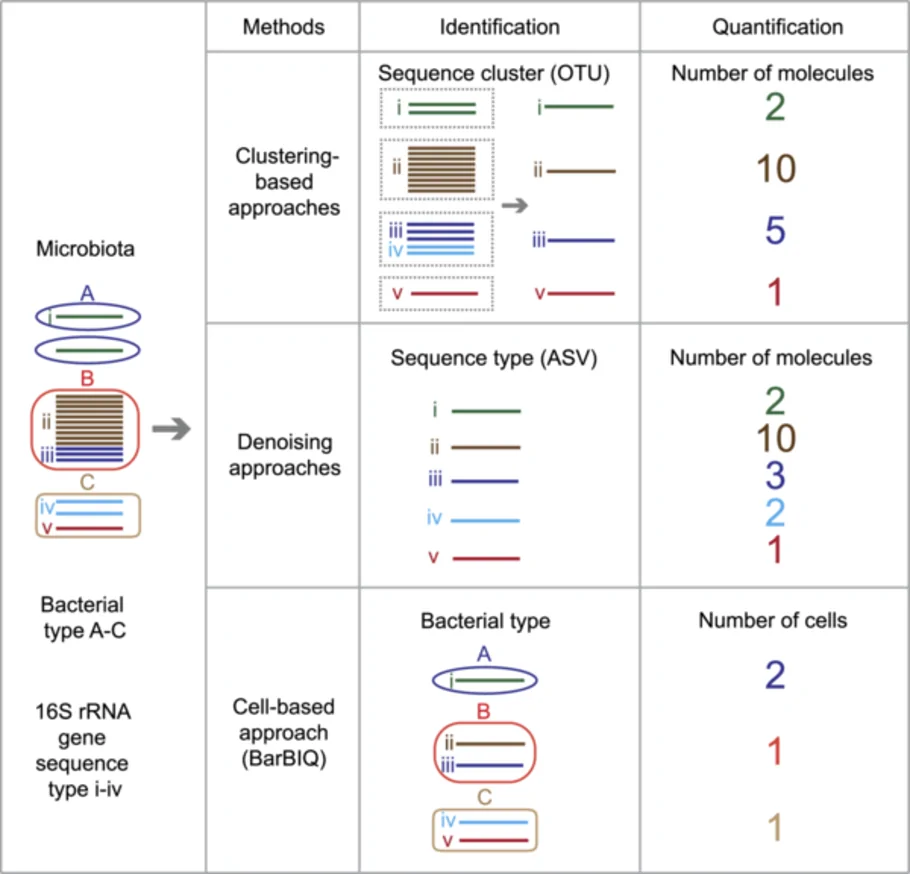
Essential differences between the representative 16S rRNA gene‐amplicon sequencing approaches. ASV, amplicon sequence variants; OTU, operational taxonomic unit; rRNA, ribosomal RNA.

Second, to correct the sequencing and amplification errors (including chimeras) in a 16S rRNA gene sequencing dataset obtained from a bulk sequencing experiment, denoising algorithms, such as divisive amplicon denoising algorithm 2 (DADA2), which uses a computational denoising model to distinguish the correct and incorrect sequencing reads by statistical analysis based on the error rates of sequencing and amplification [44, 80], have been developed and are now integrated into QIIME2 (Table 1) [54]. The denoising approaches were first applied to the data obtained by next‐generation sequencing [37, 41, 51, 81] and further were adapted to the data obtained by single‐molecule long‐read sequencing [81]. The denoising approaches identify 16S rRNA gene sequence types by detecting errored sequences using a mathematical algorithm based on the assumption that sequencing or amplification errors in the sequencing reads are rare, and the identified sequence types are referred to as amplicon sequence variants (ASVs) (Figures 1 and 2) [80]. Although the error correction strategy of denoising approaches is efficient [44, 55, 80, 81], it is not sufficient; for example, errors are not completely corrected in DADA2, even for low‐complexity communities with only 8−20 bacterial strains [38, 81]. Notably, the ASV‐based approach identifies 16S rRNA gene sequence types, which has been performed by the 16S rRNA gene‐amplicon sequencing methods without using the clustering approaches since 1995 [40]. In addition, OTU‐based and ASV‐based tools usually generate significantly different 16S rRNA gene sequence types [82, 83].

Third, DNA molecular barcoding [84] was first applied in 16S rRNA gene‐amplicon sequencing approaches in 2013 to achieve high accuracy in sequence determination [56, 57]. In this approach, each 16S rRNA gene molecule is attached to a unique molecular barcode (i.e., a different DNA sequence) that distinguishes individual molecules even when these molecules have identical sequences, and DNA molecules linked with molecular barcodes are then amplified. Therefore, after sequencing, all reads amplified from the same 16S rRNA gene molecule can be tracked by the attached molecular barcode, and the sequencing errors in each read can be corrected by comparing the reads amplified from the same molecule, assuming that there are fewer sequences containing errors than correct sequences. Although the molecular barcoding approach can correct sequencing errors, it is powerless to address amplification errors, particularly chimeras, which also represent a major problem in 16S rRNA gene‐amplicon sequencing [49].

### Recent progress in the identification of 16S rRNA genes for expanded applications

In the past few years, 16S rRNA gene‐amplicon sequencing approaches for the identification of 16S rRNA gene sequence types have been improved in the following aspects, including DNA extraction, primer selection, DNA amplification, and bioinformatic tools, which expanded their applications. First, specific DNA extraction protocols were developed or suggested for low‐bacterial‐biomass samples, such as biopsies, blood, tissues, tissue swabs, and lavages [85, 86, 87, 88, 89, 90]. The 16S rRNA gene DNA enrichment method was also developed for extremely low‐bacterial‐biomass samples, such as clinical specimens and tumor tissues [91, 92].

Second, the well‐established primer sets targeting different regions of the 16S rRNA genes (V1−V2, V1−V3, V3−V4, V4, V4−V5, V5−V7, V6−V8, and V7−V9) were compared, and the results suggested that an appropriate targeted region is essential for each study [93, 94, 95, 96]. More primer sets were investigated by mapping them to known 16S rRNA genes in the database [97, 98]. Furthermore, to cover longer genomic regions for higher taxonomic resolution, primer sets that amplify the full length of 16S rRNA genes and 16S rRNA genes with adjacent genomic contexts were used [81, 99, 100]. In addition to these “universal” primers, primer sets for the unique bacterial groups were also designed by optimizing various parameters, including primer melting temperature, GC content, and the Max Poly‐X [101, 102, 103, 104]. The newly designed primers expanded the application of 16S rRNA gene‐amplicon sequencing approaches to many special samples, such as arctic microbial communities, respiratory microbiotas, equine gut microbiota, freshwater microbiota, and oceanic trench sediment microbiotas [94, 96, 99, 101, 104]. Importantly, this technical advance leads to the identification of novel taxonomic groups, such as Asgard archaea, Bdellovibrio, and Brocadiales anammox bacteria [101, 102, 103].

Third, the amplification methods to generate sequencing libraries were improved. In next‐generation sequencing‐based approaches, amplification protocols were investigated to obtain high efficiency of specific amplification [105, 106]. In addition, droplet digital PCR was applied to increase the amplification efficiency from a low‐bacterial‐biomass sample [107]. This improved method achieved the analysis of 16S rRNA genes in very low concentrated DNA samples, water body [107]. Furthermore, a synthetic long‐read technology, LoopSeq, was applied to the 16S rRNA gene‐amplicon approach, which accurately identified full‐length 16S rRNA gene sequences [108]. This accurate technology distinguished strains within species from retail meat samples and identified potential foodborne pathogens [108].

Fourth, to improve the accuracy for the identification of long‐read 16S rRNA gene sequence types in single‐molecule sequencing platforms, new bioinformatic tools that detect sequencing errors using a mathematical algorithm, such as MetaMaps [59], Emu [60], NanoCLUST [61], and 16S full‐length amplicon sequencing data analysis software (16S‐FASAS) [62] were developed (Table 1). The 16S rRNA gene‐amplicon sequencing approach improved by Emu accomplished the same task as a whole‐genome shotgun metagenomic approach that classified the bacterial communities of 12 vaginal swabs into six so‐called “community state types” [60].

## 4 IDENTIFICATION OF BACTERIAL TYPES (cOTUs) BASED ON 16S rRNA GENES

The identification of bacterial types in a microbiota is a basis for understanding the function of the microbiota [109]. A common approach for the identification in the past decades is that many organisms are grown by culturing from a single bacterium, followed by sequencing of their genomes or 16S rRNA genes [110]. However, this approach is highly dependent on the success of bacterial culturing. Unfortunately, a large number of bacterial species (e.g., from the gut) remain unculturable [111]. Furthermore, this approach has a low throughput, which limits the analysis of microbiota characteristics.

As we discussed above, the culture‐independent approach of 16S rRNA gene‐amplicon sequencing is a high‐throughput method. However, since many bacteria have multiple 16S rRNA sequence types in their genome [14, 48], the 16S rRNA gene types identified via conventional methods often do not represent bacterial types.

To identify bacterial types from the microbiota in a high‐throughput manner, a cell‐based 16S rRNA gene‐amplicon sequencing method BarBIQ was developed recently (Table 1) [38, 39]. BarBIQ is a high‐throughput method and can determine the bacterial types of >10^5^ cells in a single experiment by determining 16S rRNA gene sequences from individual bacteria with single‐base accuracy and resolution. The 16S rRNA gene‐based bacterial type identified by BarBIQ is defined as a cell‐based operational taxonomic unit (cOTU) (Figure 1). The term cOTU is essentially different from the terms OTU and ASV, which are used to define 16S rRNA gene types (Figure 2). Notably, for unicellular organisms, the cOTU concept is the same as that of the original “OTU” of traditional taxonomy, which represents a group of organisms defined by intrinsic phenotypic similarity [76, 77].

## 5 QUANTIFICATION OF 16S rRNA GENE MOLECULES

The quantification of the cells of each bacterial type in the microbiota is another main goal of 16S rRNA gene‐amplicon sequencing approaches. However, most methods count the abundances of 16S rRNA genes, not the number of bacterial cells (Figure 2), since different bacteria have distinct copy numbers of 16S rRNA genes in their genomes (from 1 to 15 copies) [16, 112, 113, 114]. In this section, we summarize the approaches used to count the number of amplified 16S rRNA gene molecules (sequencing reads). Then, we discuss the approaches that count the number of intact 16S rRNA gene molecules (before amplification).

### Quantification of amplified 16S rRNA gene molecules (sequencing reads)

In 16S rRNA gene‐amplicon sequencing approaches, each 16S rRNA gene molecule is amplified to produce multiple DNA molecules. After the sequencing reads are clustered into OTUs or assigned to ASVs, the number of amplified 16S rRNA gene molecules (sequencing reads) for each defined OTU or ASV is counted (Table 1). The relative number of sequencing reads, or relative abundance, of each OTU or ASV, is obtained by normalization to the total detected number of sequencing reads in a given sample. However, the amplification is biased; different intact gene molecules (the molecules before amplification) may be amplified with distinct efficiencies due to their unique 16S rRNA gene sequences [115]. This leads to the relative abundances of the amplified DNA molecules of each gene type being poorly correlated with the relative abundances of the intact gene molecules [115]. Of note, the use of different bioinformatic tools might affect the determination of relative 16S rRNA gene abundances [116].

### Quantification of intact 16S rRNA gene molecules

To quantify the number of 16S rRNA gene molecules by removing the effect of amplification bias and noise, molecular barcoding approaches were developed [56, 57, 58]. As we mentioned above, each 16S rRNA gene is attached to a unique molecular barcode and amplified, and the sequencing reads can be traced back to the original 16S rRNA gene molecule based on the attached molecular barcode sequences. Therefore, the count of intact (i.e., before amplification) 16S rRNA gene molecules for each gene type can be determined by the number of molecular barcode types (Table 1) [58]. The relative counts of 16S rRNA gene molecules can be obtained by normalization to the total detected number of 16S rRNA gene molecules.

To determine the absolute counts of 16S rRNA gene molecules, theoretical prediction with molecular barcoding has been applied [58]. In this prediction approach, the authors assume that amplification using PCR is a stochastic process and estimate the absolute number of 16S rRNA gene molecules for each detected gene type in the original pool from its measured number of 16S rRNA gene molecules by Poisson statistics calculation.

In recent years, several studies [117, 118, 119, 120, 121, 122, 123, 124] have tried to use the spike‐in, total concentration, or bias estimation to normalize the relative abundance of OTUs. However, these approaches have yet to be combined with the molecular barcoding approach. Therefore, it is difficult to accurately determine the absolute counts of 16S rRNA gene molecules since their relative abundance is determined by the relative counts of sequencing reads, which depend on amplification bias and noise, not the counts of intact 16S rRNA gene molecules before amplification [58, 115].

## 6 ABSOLUTE QUANTIFICATION OF BACTERIAL CELLS

Determining the number of cells for each bacterial type is also a basis for characterizing the microbiota [36, 125]. However, it is difficult to measure and compare the number of bacterial cells by counting 16S rRNA gene molecules because different bacteria have distinct copy numbers of 16S rRNA genes in their genomes (from 1 to 15 copies) [112, 113, 114]. Indeed, Pace et al. made a similar observation in their report of the first 16S rRNA gene sequencing method for the bacterial community [16]. In the past decade, some researchers have tried to solve this problem using bioinformatic approaches [113, 114]. The main idea is to correct the number of 16S rRNA genes based on the copy number of 16S rRNA genes per cell. Since the 16S rRNA gene copy numbers of many bacteria are unknown [126], several software programs have been developed and the newest software indeed has increased the accuracy of copy number estimation [113, 127, 128, 129, 130, 131]. However, since these software predict the copy numbers of unknown bacteria by assuming that bacteria with similar 16S rRNA gene sequences in the same taxa have the same copy numbers, which may not be true for all bacteria [132], accurate prediction using these bioinformatic approaches has not yet been achieved [113, 126].

To experimentally count bacterial cells, the single‐cell BarBIQ approach was developed (Table 1) [38, 39]. BarBIQ counted the relative cell number of each identified bacterial type by attaching cellular barcodes to the amplified 16S rRNA gene molecules from each single bacterial cell in a high‐throughput manner. Then, the absolute cell number per unit weight or volume for the identified bacterial type (cOTU) in the sample is obtained by normalizing the relative cell number using the total cell number per unit weight or volume of the same sample measured separately by droplet digital PCR.

Studies have attempted to determine the absolute number of bacterial cells for each bacterial taxon by normalizing the relative counts of 16S rRNA sequencing reads of OTUs using the absolute total count of bacterial cells (determined by flow cytometric enumeration) of a microbiota sample [36, 133] or an internal control (previously measured number of bacterial cells or yeast cells containing the 16S rRNA gene) [124, 134]. In these studies, the relative counts of OTUs were corrected based on the 16S rRNA gene copy number per cell. However, as we discussed above, OTUs do not always represent bacterial types [14, 38, 48], and 16S rRNA gene copy number correction using current methods is not reliable [113, 126]. Therefore, determining absolute cell numbers using these approaches is not accurate.

## 7 CONCLUSION AND FUTURE PERSPECTIVES

The recent developments of 16S rRNA gene‐amplicon sequencing enable the identification and quantification of bacteria cells and full‐length 16S rRNA genes, which leads to a new era of microbiota characterization. However, 16S rRNA gene‐amplicon sequencing has limitations. Since 16S rRNA gene‐amplicon sequencing determines only one gene (i.e., 16S rRNA gene) for each bacterium, it is difficult to classify the bacteria into groups with a high taxonomic resolution, for example, strains. This issue can be addressed by employing multilocus sequence analysis, for example, analysis of 16S‐ITS‐23S rRNA operons instead of the 16S rRNA gene alone. Furthermore, using different conditions, such as temperature and time, during the sample collection and storage process may change the composition of microbiota in the sample or measured results due to bacteria growth or cell lysis. Therefore, it is important to develop a standardized sampling and storage protocol across various sample types [135, 136]. In addition, for samples with low bacteria biomass and a lot of host DNA, the removal of host contamination is essential in 16S rRNA gene‐amplicon sequencing. Although the current methods to remove host contamination are effective, these methods include complex procedures that need to be designed specifically for each sample [137, 138]. Therefore, universal and simple methods to easily handle low‐bacteria‐biomass samples are desired.

**Figure 3:**
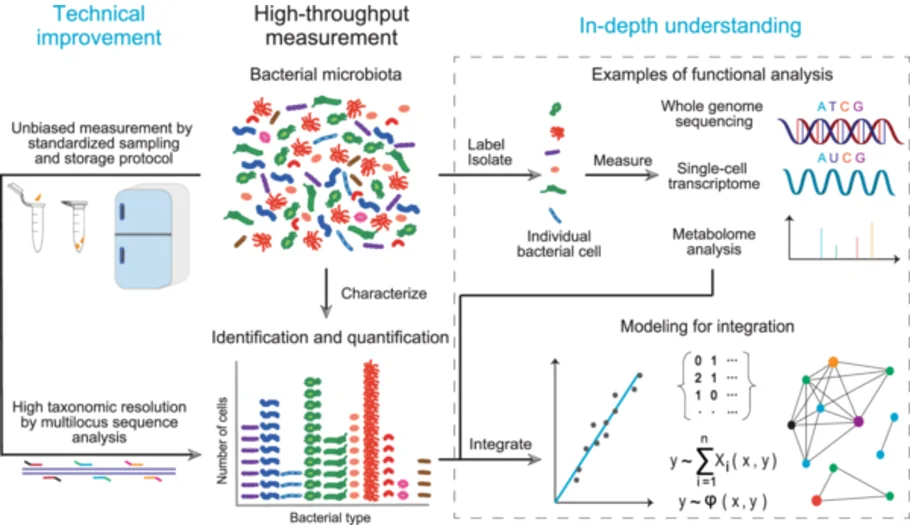
Perspective for an in‐depth understanding of bacterial microbiotas.

One of the ideal genomic measurement strategies for the characterization of bacterial microbiota is single‐cell whole‐genome sequencing [139, 140, 141] for all (many) individual bacterial cells in the microbiota, which allows a quantitative and functional understanding of the microbiota based on the number of cells and corresponding estimated genes with strain‐level resolution. However, the limited capacity of the sequencer and the high cost of this method of sequencing prohibit its use. Hence, the following hypothesis emerges: if the 16S rRNA sequence of an identified bacterial type can be mapped to a 16S rRNA sequence obtained from sequencing of the whole genome, many bacteria in the microbiota defined by whole‐genome sequences can be measured. However, this is almost impossible because the database of bacterial whole genomes is still limited [109]. A useful approach may be the combination of BarBIQ that enables the identification of many bacterial cells using a particular gene(s) and the following recently reported method [139] that enables single‐cell whole‐genome sequencing for a targeted bacterial cell that has a particular gene(s) or sequence(s) (Figure 3): identification of the 16S rRNA sequence(s) of a featured bacterial type and determination of the whole genome of the bacteria. This approach results in the ideal measurement described above. Importantly, this approach is advantageous, especially for newly identified minor bacterial types, because simple single‐cell whole‐genome sequencing of randomly selected cells (i.e., without target information) may not work for rare bacterial types.

Furthermore, targeted bacterial cells characterized by high‐throughput measurements may be isolated alive using a recently reported method [142]. This approach allows for the performance of functional analyses, such as secretion molecule measurement [143] and single‐cell transcriptome analysis [144], although some analyses may need to be improved to have higher sensitivities or may require proliferation of the isolated cells.

Once these measurements are successfully performed, mathematical modeling [145] based on the number of cells and their (estimated) functions would be helpful for understanding and predicting the overall behavior of the bacterial microbiota, including time‐course changes (Figure 3). This whole process may provide a useful tool for application in multiple fields, such as medicine and agriculture, and bacterial microbiota behavior may be predicted under conditions such as fecal transplantation [146] and agrochemical treatment [147].

Collectively, recently developed high‐throughput approaches for the accurate identification and quantification of bacterial cells using 16S rRNA‐amplicon sequencing of microbiotas will pave the way for a new era in which integration with the functional analyses described above may provide an in‐depth understanding of the bacterial microbiota.

## AUTHOR CONTRIBUTIONS

**Jianshi Jin**: Funding acquisition; conceptualization; writing—original draft; writing—review and editing; visualization; supervision. **Xiongduo Liu**: Writing—original draft; writing—review and editing; visualization. **Katsuyuki Shiroguchi**: Conceptualization; writing—original draft; writing—review and editing; project administration; supervision; visualization.

## CONFLICT OF INTEREST STATEMENT

The authors declare no conflict of interest.

## ETHICS STATEMENT

No animals or humans were involved in this study.

## Data Availability

Data sharing is not applicable to this article as no new data were created or analyzed in this study. Supplementary materials (graphical abstract and Chinese translated version) may be found in the online DOI or iMeta Science http://www.imeta.science/imetaomics/.
